# The effect of literature experience of language education teachers on their professional goals and expectations

**DOI:** 10.3389/fpsyg.2025.1702445

**Published:** 2025-12-09

**Authors:** Yasemin Albayrak, Nurullah Aydın, Sıddık Bakır

**Affiliations:** Department of Turkish Education, Kazım Karabekir Education Faculty, Atatürk University, Erzurum, Türkiye

**Keywords:** language education, use of literature, career goals, reading habit, critical thinking

## Abstract

This study examines how language teachers in Turkey develop personal and professional relationships with literature and how these relationships influence their teaching goals and classroom practices. Qualitative data were collected through semi-structured interviews with 30 middle school Turkish language teachers in Erzurum, and the data were analyzed using content analysis. The findings show that personal reading habits, perceived literary competence, and cultural orientations shape teachers’ use of literature as an instructional tool. Teachers reported that literary texts support the development of language skills, critical thinking, and creativity, while also noting challenges such as selecting appropriate texts, limited instructional time, and differences in student readiness. The results further indicate that teachers’ cultural backgrounds and personal values significantly influence their text choices and pedagogical orientations. Overall, the study highlights the role of literature in fostering both cognitive and cultural development and recommends that teacher education programs promote regular reading habits and systematic approaches to strengthen literary competence.

## 1 Introduction

There is a growing consensus among researchers, teacher educators, and policymakers that prospective teachers’ personal connections with literature should be linked to their development of a professional identity (Beauchamp and Thomas, 2009; Martel, 2015; Calafato, 2024; Hauber-Özer et al., 2025). This approach allows language teachers to enhance their pedagogical sensitivity and engage in more meaningful interactions with students. In this context, Nussbaum (2016) emphasizes the power of literature to foster empathy, ethical sensitivity, and cultural awareness in individuals, stressing that teacher education should be enriched with literary content that cultivates these human values. Farrell (2016), on the other hand, argues that teachers’ professional growth should be supported not only by technical knowledge but also by humanistic and aesthetic values. This holistic perspective encourages a human-centered teaching approach that boosts teacher candidates’ professional motivation and awareness of social responsibility. Recent meta-analytic evidence on reading-focused professional development further shows that well-designed PD can substantially enhance teachers’ instructional knowledge and skills (Filderman et al., 2025), highlighting the need to connect such professional learning opportunities with teachers’ personal literary trajectories.

This holistic approach supports teacher candidates’ development of both professional and human values through literature and enhances their meaning-making processes by fostering aesthetic sensitivity and deeper understanding. Literature offers a multi-layered experience that activates individuals’ aesthetic, emotional, and cognitive faculties at the same time. Readers not only connect with the linguistic meaning of literary texts but also examine their emotional, ethical, and cultural aspects. This engagement is vital in shaping teachers’ reading habits, professional identities, and teaching methods. Over time, it also assists teachers in integrating literary texts into their classrooms and nurturing students’ aesthetic and emotional awareness. In this way, literary texts act as tools that both enrich teachers’ personal reading experiences and establish meaningful connections with their students (Tilmatine et al., 2024; Sprang, 2017).

The fact that literature plays such a central role in the intellectual, emotional, and ethical development of teachers raises questions about how it influences their professional orientation, goals, and expectations. This study examines how language teachers’ personal and professional experiences with literature impact their professional identity, teaching methods, and career aspirations within this context. Literature is seen not just as a reading activity for teachers but as an experiential field that nurtures identity growth, value development, and meaning-making processes. Therefore, the research focuses on analyzing teachers’ interactions with literary texts across cognitive, emotional, and ethical dimensions to understand how these experiences influence their professional awareness and teaching practices.

This study aims to explain, on a conceptual level, how literature transforms teachers’ thinking, motivations for teaching, and classroom decisions. By highlighting the connection between teachers’ personal literary experiences and their professional goals and expectations, it shows that literature offers a learning space that encourages growth in language education both personally and pedagogically. Consequently, the research promotes a reevaluation of the ethical, cultural, and cognitive foundations of the teaching profession from a literature-centered perspective.

## 2 Literature review

### 2.1 A framework of professional goals and expectations based on the literary experiences of language teachers

Language teachers’ professional goals and expectations are shaped by a multi-layered framework influenced not only by institutional standards but also by their personal literary experiences, pedagogical values, sources of motivation, and perceptions of professional competence. This framework is based on the conceptual model presented below. Table 1 illustrates how this complex structure works and how literary experiences can significantly influence the development of teacher competence. Gao et al. (2022) highlight that teachers’ professional orientations extend beyond cognitive skills and are equally shaped by emotional tendencies, pedagogical values, and perceptions of self-efficacy. Therefore, teachers’ reading habits, attitudes toward literature, and professional motivations become key factors that contribute to both personal and professional growth.

**Table 1:** Process model of the transition from literary experience to teacher competence.

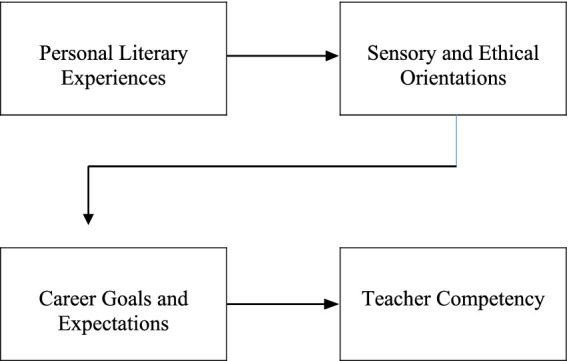

This framework emphasizes the importance of examining teachers’ literary experiences from a multidimensional perspective that integrates cognitive, affective, and professional dimensions, rather than treating them as isolated variables. Most previous studies in language education tend to evaluate these elements separately and often neglect how they interact to shape teachers’ identities and pedagogical orientations. Research examining the relationship between teachers’ career goals and their pedagogical knowledge acquisition has shown that professional development and education-focused goals play an important role in the development of higher-order cognitive processes (Klemenz and König, 2024). The components of the framework are discussed in detail under the following subheadings.

### 2.2 Literature-related experiences, motivations, and professional expectations of language teachers

Literary experiences involve engaging with texts that hold personal meaning and evoke cognitive, emotional, and educational responses. These experiences are closely connected to teachers’ reading skills, motivation, self-efficacy, and teaching approaches. While Guthrie and Wigfield (2000) highlight that reading engagement and motivation arise from meaningful interaction with texts, Guthrie et al. (2013) demonstrate that both cognitive and emotional factors greatly influence teaching effectiveness. Similarly, Calafato (2024) discovered that teachers’ reading habits and creativity strongly predicted their literary competence and affected how they viewed literature and integrated it into their teaching. These insights indicate that emotional attachment to reading plays a critical role in developing literary sensitivity. Simultaneously, this attachment also helps shape teachers’ professional identity. Recent evidence also demonstrates that reading behaviors are strongly shaped by motivational and affective mechanisms. For instance, Zhang et al. (2025) found that digital reading literacy enhances social reading motivation, which in turn significantly predicts actual reading behavior. These results underscore that reading engagement is not solely a cognitive process but also deeply rooted in emotional and motivational dynamics. Zhu et al. (2022) showed that teacher trust and commitment mediate the effect of school leadership on students’ reading literacy, indicating that teachers’ emotional orientations shape both their engagement with literature and the broader learning environment.

Many studies rely on teachers’ personal narratives and often focus on general beliefs about the challenges, benefits, or enjoyment of literature. However, relatively few research works explore how these beliefs influence specific teaching practices. In addition to these overall attitudes, teachers also hold explicitly value-based views on literature that connect their belief systems to classroom practices (Abukari, 2014). Some see literature as reinforcing cultural identity and moral values, while others consider it a resource that promotes global citizenship and intercultural understanding. This variety demonstrates that teachers’ literary perspectives also influence their teaching methods.

Empirical research also supports this perspective. Teachers’ reading orientations and interpretation paradigms are shown to strengthen critical pedagogy and increase classroom participation (Zyngier and Fialho, 2010). Regmi (2021) identifies four main approaches to teaching literature: text-focused, language-focused, reader-response-based, and critical. Each approach influences teacher-student interactions differently. Penny (2011) notes that applying stylistics and close reading techniques can improve linguistic awareness by analyzing grammatical and discursive features. Together, these approaches demonstrate that teachers can help students achieve both linguistic accuracy and empathetic understanding by integrating technical, aesthetic, and emotional aspects into their teaching. The classroom implications of these orientations are also reflected in teaching strategies.

Teaching strategies differ in complexity and accessibility. While some methods enhance students’ text comprehension, multimodal approaches promote visual and critical literacy. According to Regmi (2021), teachers modify these strategies to suit their class needs and improve learning outcomes. However, exam-focused curricula, crowded classrooms, time constraints, and varying proficiency levels limit teachers’ ability to implement this diversity (Fikray and Habil, 2022). Additionally, the emotional or motivational factors influencing students’ responses to these methods have not been thoroughly examined in the literature. Focusing more on these aspects could help deepen understanding of how students connect with texts across different cultural and linguistic backgrounds.

Beyond pedagogical beliefs, teachers’ literary skills are influenced by aesthetic sensitivity, emotional disposition, personality traits, and out-of-school reading habits. These interconnected factors create a multidimensional framework where cognitive, affective, and personal elements intersect and shape professional growth (Gutierrez, 2004). Supporting this, Yang et al. (2018) found that reading beliefs and self-efficacy strongly predict teachers’ motivation to incorporate literature-based activities into their teaching. Teachers who see reading as a means of personal enrichment tend to adopt more interactive, student-centered methods, while those with lower reading self-efficacy rely more on exam-focused or translation-based approaches. These findings show that teachers’ belief systems and perceived competence influence how they engage with literature.

Generally, exploring teachers’ experiences and motivations regarding literature not only clarifies their professional goals but also shows how personal reading interests influence their teaching methods-especially in non-English-speaking environments, where cultural and language differences require more adaptation and reflection. These insights offer a valuable understanding of how teachers’ literary backgrounds connect to their teaching strategies.

### 2.3 Professional experience, individual reading, and classroom practice

For literature to be effectively integrated into language education, both teachers and students need a certain level of literary competence. This competence includes skills such as recognizing formal and aesthetic features (aesthetic and stylistic), empathizing with characters, inferring meaning, and analyzing cultural or discursive representations. Research highlights that teachers’ literary knowledge and reading experience are essential for guiding students in developing these skills (Calafato, 2024). Besides technical skills, teachers’ emotional connections to texts and their ability to relate literature to life experiences also play a vital role in fostering genuine and meaningful classroom engagement.

International standards have also outlined this complex structure of teachers’ literary competence. The updated CEFR descriptors define literary competence through analytical, critical, and personal response dimensions, emphasizing literature as a lifelong pursuit of reading and aesthetic appreciation. Alter and Ratheiser (2019) identify four key components of literary proficiency aligned with these descriptors and demonstrate their practical classroom use with examples. CEFR stresses that learners should be able to respond personally to texts, interpret linguistic and thematic meanings, analyze literary devices, and make critical judgments about technique and content. These descriptors show the balance between analytical accuracy and personal interpretation, illustrating that emotional connection and critical analysis mutually enhance understanding of literature. This view on literary proficiency is also supported by research exploring both emotional and cognitive aspects.

Supporting this integrative perspective, de la Maya Retamar and López-Pérez (2021) found that EFL teacher candidates’ literary competence is closely linked to their emotional intelligence. Teachers who interact with literature emotionally and empathetically interpret texts more profoundly and use this awareness in their teaching methods. These findings emphasize that literary understanding involves both cognitive and emotional components, aligning with the CEFR’s emphasis on combining analytical precision with emotional engagement. Additionally, the data shows that teachers’ literary and emotional skills are essential for understanding how similar abilities develop in students.

Despite the increasing interest in improving students’ literary skills in their learning environments (Ghosn, 2013; Paran, 2008; de la Maya Retamar et al., 2024), research on teachers’ own literary abilities remains limited. Neno et al. (2022) found that teachers’ reading habits directly impact students’ literacy development and influence overall classroom instruction quality: “Teachers’ lack of reading habits limits students’ opportunities to receive quality literacy education.” Similarly, Nai et al. (2022) discovered that English teachers with weak personal reading habits and a focus on translation tend to foster significantly lower reading comprehension among students, averaging only 51%. Their study shows that teachers’ limited exposure to diverse literary texts and their tendency to read mainly for work or entertainment hinder the development of a sustainable reading culture in the classroom. Therefore, teachers’ personal reading identities—both emotionally and habitually—are crucial to their ability to serve as models of literary engagement for students. Studies indicate that people’s reading habits and genre preferences show long-term stability and are largely shaped by environmental factors such as the availability of reading materials and supportive literacy spaces (Le Gac and Çakar, 2025).

Other studies also demonstrate a positive link between teachers’ reading habits and their instructional activities. McKool and Gespass (2009) found that teachers who read at least 30 min daily used a wider variety of effective reading strategies and increased student motivation more. Similarly, an extensive national study by Morrison et al. (1998) involving 1,874 elementary school teachers found that active readers implemented recommended literacy practices—such as read-aloud sessions, book discussions, and sustained silent reading—more often than non-active readers. These findings suggest that personal reading engagement improves teachers’ pedagogical skills and enhances their ability to serve as authentic role models for students’ reading.

Literary proficiency depends on how often people read; however, recent studies indicate that the quality of teachers’ reading experiences is a stronger predictor of their depth of pedagogy. Míguez Álvarez et al. (2023) found that many teacher candidates prefer popular commercial stories over works that are educationally or aesthetically challenging. This restricts their interpretive range and affects how they teach literature. Additionally, little attention has been given to how cultural background, access to literary materials, and emotional reading experiences influence teachers’ interpretations and teaching choices, especially in non-English-speaking settings. Addressing this gap will significantly improve a global and context-aware understanding of literary teaching. Trends along similar lines also encourage new discussions about the importance of creativity, innovation, and intrinsic motivation in teachers’ classroom practices.

It has also been shown that teachers’ creativity influences student achievement through intrinsic motivation (Du et al., 2019). Although the link between creativity and school performance is generally modest, it varies by educational level and assessment type, with a stronger connection observed in standardized tests (Gajda, 2016). Defined as the ability to generate original ideas through diverse ways of thinking, creativity enables flexible, innovative teaching methods (Allegri, 2021). Calafato (2024) found that teachers’ creativity and reading habits significantly predicted their literary proficiency, and that creative traits were closely linked to interpretation and analytical skills. In this context, creativity and literary engagement act as mutually reinforcing processes: creative reading fuels innovative teaching, while creative teaching maintains teachers’ emotional connection to literature. Recent research by Wang et al. (2025) further supports this by showing, through structural modeling, that teachers’ emotional attachment mediates the relationship between personal engagement and pedagogical innovation. Their findings demonstrate that teachers who are emotionally attached to reading are more likely to adopt creative, student-centered pedagogies, revealing how this attachment can directly lead to innovative classroom practices. Therefore, teachers’ personal reading experiences and creative thinking skills are two essential, complementary elements for sustaining literature-based teaching methods.

### 2.4 Research questions

In Turkey, few studies have examined the personal and professional relationships that language teachers develop with literature, their perceptions of literary competence, and how these perceptions influence their teaching methods. As a result, our understanding of how teachers’ ability to use literature as a resource in their lessons relates to their personal literary experiences and creative thinking skills remains limited. This study aims to address this gap by exploring the following questions:

1. How do teachers perceive the influence of their personal experiences with literature on their professional goals as language educators?
2. How does literature influence language teachers’ expectations regarding students’ language proficiency and academic achievement?
3. How does language teachers’ interest in literature contribute to their professional development, particularly in terms of language competence and teaching effectiveness?
4. What are the advantages and challenges experienced by language teachers when using literary texts as teaching materials?
5. How do language teachers perceive the contribution of literature use to the development of students’ critical thinking and creativity skills?

## 3 Methods

### 3.1 Context and participants

The study was carried out in several middle schools in Erzurum, Turkey, where Turkish, English, Arabic, and German are taught as foreign languages. The participants included 30 in-service language teachers (18 female, 12 male), who were intentionally selected to ensure they had relevant professional experience and ongoing engagement with literature. The inclusion criteria specified that participants (1) had at least 3 years of teaching experience, (2) were currently employed as foreign language teachers at the middle school level, and (3) volunteered to participate after being informed about the study’s aims, scope, and ethical standards.

The participants’ ages ranged from 28 to 54 years (M = 41.8, Mdn = 40.0, SD = 7.75). Most held bachelor’s degrees (*n* = 22, 73.3%), while eight teachers (26.7%) held master’s degrees. Teaching experience varied from 4 to 34 years (M = 17.1, Mdn = 13.5, SD = 8.68). In terms of subject specialization, 43.3% (*n* = 13) taught Turkish, 23.3% (*n* = 7) taught English, 16.7% (*n* = 5) taught Arabic, and 16.7% (*n* = 5) taught German. Participants reported spending between 0.75 and 20 h per week on reading activities (M = 6.19, Mdn = 5.00, SD = 4.66).

These languages are not the first languages of students in Erzurum; rather, they are learned primarily for communicative and academic purposes (Karaata, 2008). Although English has long been a compulsory subject in the national curriculum, only a small proportion of Turkish adults demonstrate functional fluency in the language (British Council and TEPAV, 2015).

Participant recruitment followed a thorough and ethically sound process. A comprehensive list of middle schools in Erzurum was obtained from official education portals, and school administrators were contacted to identify teachers who met the inclusion criteria. Each potential participant received an invitation letter explaining the study’s purpose, ethical assurances, and confidentiality measures. Teachers who volunteered contacted the researcher directly. To ensure anonymity, all participants were assigned alphanumeric codes (P01–P30). The relevant institutional ethics committee granted ethical approval for the study.

### 3.2 Data collection

The data were gathered through semi-structured interviews aimed at exploring language teachers’ professional and personal connections with literature.

Semi-structured interviewing was selected because it ensures consistency across participants while allowing for an in-depth exploration of individual experiences (Kallio et al., 2016).

The interview protocol was developed and refined across multiple stages. First, existing studies on teachers’ literary engagement, professional identity, and pedagogical beliefs were thoroughly reviewed (Calafato, 2024; Paran, 2008; Gao et al., 2024). Based on this review, an initial set of 5 open-ended questions was created. These questions were then assessed by two language education experts for clarity, alignment with the research, and appropriateness for the context. After receiving their feedback, the questions were revised to better suit the Turkish educational setting; for example, the question “How do you use literary texts in your lessons?” was expanded with sub-questions on text selection, student levels, and classroom practices.

To evaluate the clarity and usefulness of the questions, pilot interviews were conducted with two teachers. Based on their feedback, several follow-up questions were revised to elicit more detailed and clearer responses. The insights gained from the pilot phase helped finalize the interview schedule. As a result, the final version included seven thematic sections covering different aspects of teachers’ engagement with literature. These sections directly relate to the seven main themes (T1–T7) outlined in the Results section.

1. Impact on profession (T1): Questions examined how teachers’ personal reading histories and literary experiences shape their professional choices, goals, and motivation for teaching.
2. Use of literary texts (T2): Participants were asked how they incorporate literary texts into instruction, their perceived benefits, and the challenges they face.
3. Literary heritage (T3): Items examine how teachers’ accumulation of literary knowledge and background contributes to classroom effectiveness and pedagogical creativity.
4. Cognitive development (T4): Questions highlighting the role of literature in improving students’ higher-order thinking skills, particularly critical thinking and creativity.
5. Literary sensitivity (T5): This section discussed teachers’ perspectives on literature’s role in enhancing students’ aesthetic appreciation and emotional awareness.
6. Language skills (T6): Questions evaluated teachers’ perceptions of how literary texts aid the development of linguistic competence, vocabulary, and communication skills.
7. Interest and success (T7): Items explored teachers’ opinions on how exposure to literature affects students’ motivation, engagement, and academic achievement.

Each interview lasted approximately 35 to 60 min and was conducted either in person or via an online platform, depending on participants’ availability. All interviews were audio-recorded with informed consent and later transcribed verbatim by the researcher. Follow-up prompts were used when clarification or elaboration was needed, allowing participants to share detailed perspectives.

The semi-structured interview format and thematic approach enabled participants to openly share their experiences while keeping a clear focus on the study’s main goals. The data gathered from the interviews offered detailed qualitative insights into teachers’ perceptions of the educational value of literature, their teaching methods, and personal reflections.

### 3.3 Data analysis

The data were analyzed using a process that combined qualitative thematic content analysis with descriptive statistical methods. This approach aimed to provide both interpretive insights and a clear presentation of code distribution. Quantitative data from the demographic section of the interview form were analyzed with SPSS 20 to compute descriptive statistics (frequencies, percentages, and means). These statistics were used exclusively to present participants’ background information, such as gender, teaching experience, and weekly reading time.

The primary data for the study consisted of qualitative information gathered from semi-structured interviews. These data were analyzed thematically with QDA Miner Lite software. The analysis followed the six-phase thematic analysis model proposed by Braun and Clarke (2006) to ensure systematic rigor and transparency.

#### 3.3.1 Data familiarization and transcription

All interviews were transcribed verbatim and thoroughly reviewed by the researchers. Memos were written to document initial impressions of participants’ engagement with literature, their teaching reflections, and their pedagogical decisions.

#### 3.3.2 Initial coding

Codes were generated inductively from the data without relying on predefined categories. Each meaningful segment of text was labeled to reflect the main idea expressed by participants, such as reading habits, use of literary texts, critical thinking, or language development.

#### 3.3.3 Theme generation and categorization

Similar codes were grouped into subthemes and then combined into seven main themes that reflect teachers’ professional, pedagogical, and cognitive relationships with literature:

T1: Impact on profession
T2: Use of literary texts
T3: Literary heritage
T4: Cognitive development
T5: Literary sensitivity
T6: Language skills
T7: Interest and success.

#### 3.3.4 Descriptive quantification

**Figure 1:**
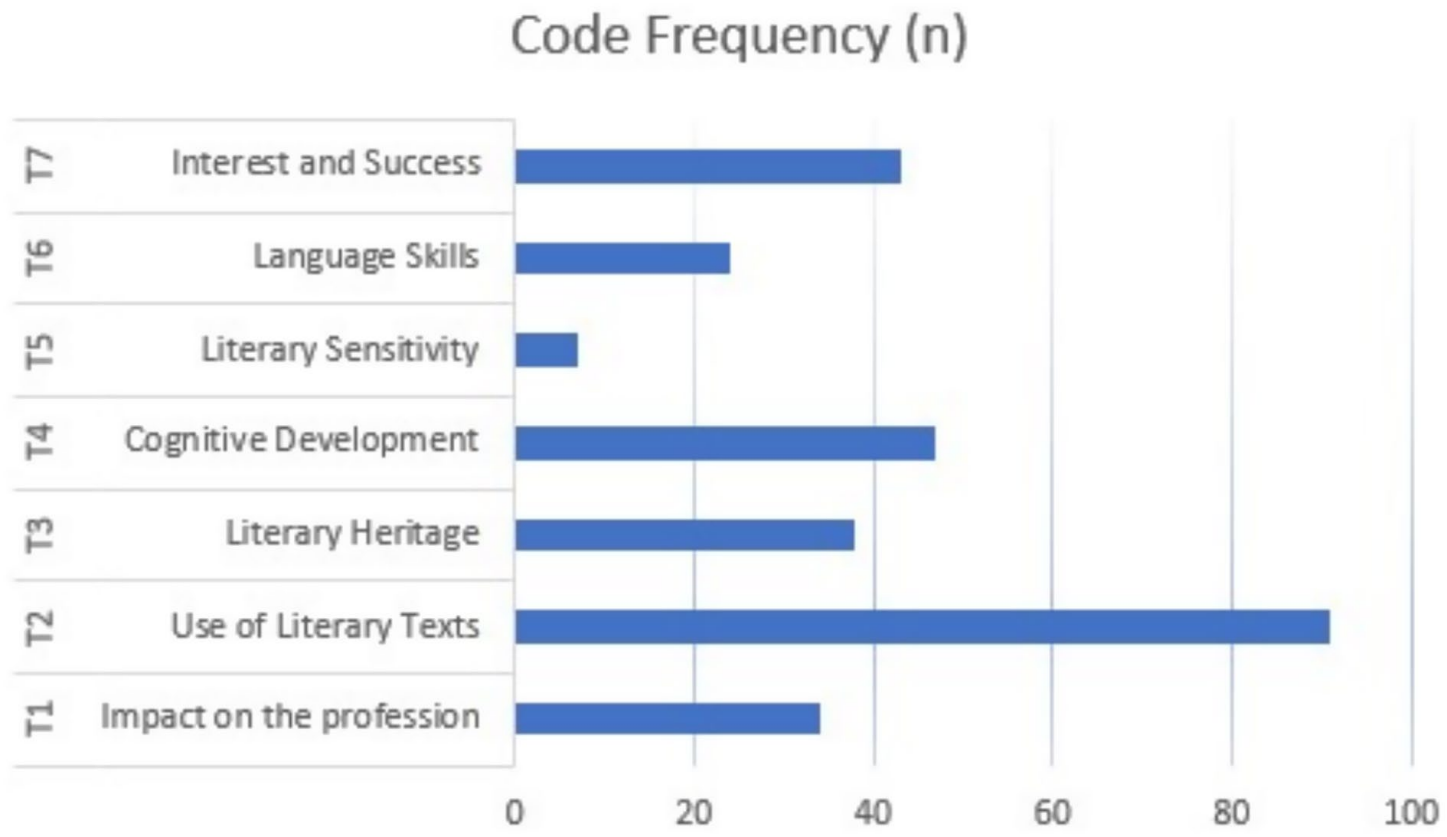
Distribution of thematic code frequencies based on participant opinions.

To visualize the distribution of participants’ views across the themes, the frequencies and percentages of codes and subthemes were exported to SPSS 20. The resulting descriptive findings were used to highlight the prominence of specific ideas and subthemes. These quantitative summaries are shown in Tables 2–8, while the overall thematic distribution is displayed in Figure 1 in the Results section.

**Table 2:** Distribution of codes and participant frequencies related to the theme of “impact on profession.”

Theme code	Theme name	Sub-theme (code)	Code frequency (*n*)	Code percentage (%)	Participant frequency (f)	Percentage of participants (%)
T1	Impact on the profession	Career-oriented experience	13	4.60%	12	40.00%
		Personal reading history	13	4.60%	12	40.00%
		Guided career decision	5	1.80%	4	13.30%
		Role model effect	3	1.10%	3	10.00%

**Table 3:** Participants’ opinions on the use of literary texts as course material.

Theme code	Theme name	Sub-theme (code)	Code frequency (*n*)	Code percentage (%)	Participant frequency (f)	Percentage of participants (%)
T2	Use of literary texts	The use of literature as material	36	12.70%	22	73.30%
		Not to be used as material	6	2.10%	6	20.00%
		Advantages	19	6.70%	16	53.30%
		Challenges	30	10.60%	21	70.00%

**Table 4:** Frequency and percentages of sub-themes and participant opinions related to the theme of “literary heritage.”

Theme code	Theme name	Sub-theme (code)	Code frequency (*n*)	Code percentage (%)	Participant frequency (f)	Percentage of participants (%)
T3	Literary heritage	Literary effects in teaching	38	13.40%	25	83.30%

**Table 5:** Frequency and percentages of participants’ views on the contribution of literary texts to cognitive development.

Theme code	Theme name	Sub-theme (code)	Code frequency (*n*)	Code percentage (%)	Participant frequency (f)	Percentage of participants (%)
T4	Cognitive development	Critical thinking	23	8.10%	18	60.00%
		Creativity	24	8.50%	20	66.70%

**Table 6:** Descriptive statistics of participants’ views on the emotional and aesthetic dimensions of literary texts.

Theme code	Theme name	Sub-theme (code)	Code frequency (*n*)	Code percentage (%)	Participant frequency (f)	Percentage of participants (%)
T5	Literary sensitivity	Sensory and aesthetic dimension	7	2.50%	6	20.00%

**Table 7:** Frequency and percentage distribution of participants’ opinions regarding the contribution of literary texts to language skills.

Theme code	Theme name	Sub-theme (code)	Code frequency (*n*)	Code percentage (%)	Participant frequency (f)	Percentage of participants (%)
T6	Language skills	Contribution to language skills	23	8.10%	17	56.70%
		No contribution to language skills	1	0.40%	1	3.30%

**Table 8:** Frequency and percentage distribution of participants’ views on the effect of literary texts on student interest and academic achievement.

Theme code	Theme name	Sub-theme (code)	Code frequency (*n*)	Code percentage (%)	Participant frequency (f)	Percentage of participants (%)
T7	Interest and success	Student interest and participation	27	9.50%	14	46.70%
		Interest-performance relationship	16	5.60%	11	36.70%

#### 3.3.5 Theme refinement and verification

The identified themes were re-evaluated for consistency, coherence, and distinctiveness. Overlapping categories were merged, and unclear codes were refined. Each theme was supported by quotes that genuinely reflected the participants’ voices.

#### 3.3.6 Reliability and credibility

Analytical reliability was improved through peer debriefing and researcher triangulation. The coding scheme was reviewed independently by two experts in language education and literature pedagogy. Discrepancies in coding were discussed until full agreement was reached, and all coding decisions and revisions were documented to maintain an audit trail. This method enhanced traceability and transparency in the process.

In the final stage, the themes closely aligned with the research questions and formed the structural foundation of the Results section. Each theme in the analysis corresponded to the thematic distributions shown in Tables 2–8 and Figure 1, ensuring that the findings from the qualitative coding and descriptive synthesis were presented in an organized and clear manner.

### 3.4 Trustworthiness and methodological rigor

The reliability and validity of the data analysis were upheld through systematic processes aligned with both the qualitative and quantitative parts of the study. Because the analysis involved thematic coding and descriptive statistical synthesis, methodological rigor was maintained by applying principles of credibility, dependability, confirmability, and transparency (Nowell et al., 2017) in an integrated manner.

To ensure credibility, thematic categories were inductively created based on the participants’ own expressions and then cross-checked with the coded data to verify accuracy. The consistency among codes, subthemes, and overarching themes (T1–T7) was assessed through repeated comparisons of the data. This process ensured that each theme genuinely reflected teachers’ perceptions and practices. For example, the relationships between literary experience and professional orientation (Table 2), the integration of literary texts into instruction (Table 3), and the development of cognitive and affective dimensions such as critical thinking, creativity, and aesthetic awareness (Tables 5,6) demonstrate this representation.

Dependability was reinforced through meticulous documentation of each stage of the analytical process. Coding choices, theme modifications, and descriptive statistics were carefully recorded to create an audit trail. Descriptive analyses of code frequency and participant percentages—displayed in Tables 2–8 and Figure 1—were re-checked in SPSS 20 and cross-verified with QDA Miner Lite results. This statistical validation improved the accuracy and reliability of the analysis process.

Confirmability was ensured through researcher triangulation. Two independent experts in applied linguistics and qualitative research reviewed the coding framework, frequency distributions, and theme assignments. Discrepancies were resolved through consensus meetings, maintaining interpretive neutrality and ensuring that all analytical decisions were data-driven. Additionally, the principle of transferability was used to improve the interpretability of the findings across different contexts.

Transferability was achieved by providing detailed descriptions of participants’ linguistic backgrounds and professional environments. These contextual details helped deepen the understanding of the thematic findings. The combination of statistical summaries and qualitative excerpts in the “Results” section (Tables 2–8; Figure 1) assists readers in tracking how perceptions of literature’s impact on teachers’ professional growth, teaching methods, and student learning were consistently illustrated throughout the dataset.

The methodological measures implemented significantly enhanced the transparency and reliability of the analysis process. Consequently, the findings accurately and comprehensively depicted the complex nature of language teachers’ professional and pedagogical relationships with literature.

## 4 Results

Table 2 presents descriptive statistics on how participants’ connections to literature influence their views on teaching as a profession. The data were analyzed through sub-themes such as reading habits, the guiding role of teachers, and the impact of literature on career choices. These themes were identified by examining codes, participant counts, and percentages. As shown in the table, participants generally see the influence of literary experiences on career paths positively. One participant’s statement supports this: “We can say that having good Turkish teachers in middle school and literature teachers in high school directly led me to this path.” (P07). However, the influence of personal reading history or teacher role models on career choice was less frequently mentioned compared to other sub-themes. Participants also demonstrated awareness of their personal reading habits and relationship with literature, as seen in P08’s statement: “Since childhood, I have been very interested in Turkish rather than literature... My relationship with books began when I was at university... Reading helped me grow, and I felt this growth in my conversations, behavior, and outlook on life.” Still, references to the role model effect were relatively limited. For example, the statement, “Some of my students are affected. They say they want to write like me... Until now, I had students who looked up to me as a role model.” (P25), illustrates this. Additionally, participants’ engagement with literature during their career decision-making seems to focus more on reading and understanding; personal reading experiences and global perspectives are more prominent than nationalist or narrow views. Frequency analyses show that the sub-themes of “personal reading history” and “career-influencing experiences” appeared more often than others in the career decision-making processes of teacher candidates. Specifically, the codes “personal reading history” and “career-oriented experience” were mentioned by 40% of participants, highlighting these as key aspects of the theme. In contrast, sub-themes such as “guided career decision” and “role model influence” were less common (13.3 and 10%, respectively). For example, a participant said, “Our choice of profession was entirely based on livelihood. I was not thinking about teaching normally.” (P29) and “It was not influenced by literature, but rather a little bit by my family... My interest in Arabic comes from my family.” (P19). These findings suggest that personal literary experiences have a statistically greater influence on career orientation.

Table 3 presents descriptive findings on how participants incorporate literary texts into their courses and their opinions on this practice. Participants stated that they effectively integrate literary texts into their lessons; specifically, they believe this approach helps students improve their language skills and that the texts offer linguistic and cultural richness. For example, P14 explained, “We already use stories, novels, fairy tales, fables, etc. in our lessons. In addition, we prepare literature-related performances and poetry recitals.” Similarly, P10 mentioned they actively include different literary genres in their lessons, stating: “If the topic is poetry, we read poems by different poets or passages from stories we have read, and we want students to read as well. In other words, we do everything we can to encourage them to read.” However, some participants noted challenges when using literary texts, due to issues like material selection, time constraints, and the texts’ appropriateness for students’ levels. It appears that the use of literary texts is limited, especially among groups with lower language proficiency. P3 explained this: “We cannot go into too much literature, poetry, or novels. The classes I take require a very advanced level of German. I cannot process much literature as material.” Another participant pointed out the difficulty at this grade level, saying, “We cannot use it in middle school; unfortunately, it is not available in elementary and middle schools.” Regarding the use of literary texts in teaching, 73.3% of participants said they find these texts contribute significantly to language instruction, while 10% said they prefer not to use literary texts. Many who recognized this benefit said literary texts enrich students’ vocabulary and foster cultural awareness. P22 described this: “I can say that almost every literary work or text directly enables them to understand and reinforce the grammar rules I am going to explain. I can achieve direct positive results.” P12 emphasized the positive effect of using American literature in class: “Actually, it was not tough; in fact, it was more helpful in focusing on the lesson... Especially when talking about black and African-American literature... when describing their past experiences and reflecting this in the books... the children’s interest and curiosity increased.” Conversely, teachers who are hesitant to use literary texts due to issues such as text selection, time constraints, or students’ varying readiness levels have also been noted. For example, P18 points out the practical challenge, stating, “Using sources other than books as sources... using other things in addition to poetry, novels, and stories is tough in this new curriculum because the Maarif model is very intensive and you can hardly ever go beyond the book.” Similarly, P23 described the challenge of time limits: “A 40-min class period may not be enough for children.” When examining participants’ views on using literary texts as teaching materials, specific sub-themes appeared more often. However, there was no clear or significant difference between these themes. Table 3 provides a detailed breakdown of participants’ opinions on using literary texts within various sub-themes.

Table 4 provides descriptive statistics on participants’ views regarding the contribution of literary knowledge to lesson delivery. The theme “Literary Accumulation” is represented by the subcode “Literary Influence in Teaching.” This sub-theme was noted 38 times, making up 13.40% of all coding. Most participants (*n* = 25, 83.3%) reported that their literary knowledge and experience help them teach more effectively. This indicates that teachers actively use their literary background in teaching and believe it improves student engagement and lesson quality. Participants’ comments suggest that using examples from literary texts makes abstract ideas easier to grasp introduces students to the beauty of language, and adds artistic depth to teaching. In this context, P20’s statement is noteworthy: “Literature develops both our vocabulary and our imagination. We can give students more accessible examples, and it helps us broaden their horizons in our classes.” Similarly, P26 highlights how literary knowledge enhances personal language skills, saying: “As I read books, I can see that there is a huge difference between my ability to express myself five years ago and my ability now. When I need to form a sentence, I can do so more easily. When I need to write a letter or share my thoughts, I can do so more comfortably.” This shows that literary heritage is not just an individual mental asset but also a valuable educational resource.

Table 5 presents data on participants’ opinions about how literary texts contribute to cognitive growth. The sub-themes “Critical Thinking” and ‘Creativity’ under the main theme of “Cognitive Development” reflect participants’ beliefs on this topic. The sub-theme “Critical Thinking” was coded 23 times overall, accounting for 8.10% of the total coding. Notably, 60% of participants (*n* = 18) said that literary texts help develop students’ critical thinking skills. In this context, P16 states that literary texts improve students’ ability to see events from different angles and analyze them critically: “From my perspective, I can see things differently, with a different perspective, a more critical eye.” Similarly, the sub-theme “Creativity” was coded 24 times (8.50%), and 66.70% of participants (*n* = 20) said that literary texts are effective in encouraging creative thinking skills. In this context, P20 states that literary texts support individuals’ mental imagery and creative imagination abilities as follows: “Let us say that when we watch a movie, we completely immerse ourselves in the scenes, and the events carry us along with the flow; but when we read a book, a novel, or a book about historical events that took place in the past, we visualize those events in our minds. They come to life in our minds, and we give the characters we read about a form and shape in our minds.”

Table 6 shows statistical data on participants’ views about the emotional and aesthetic impacts of literary texts. The sub-theme “Emotional and Aesthetic Dimension” within the broader theme “Literary Sensitivity” was coded seven times (2.50%), and six participants (20.00%) shared opinions related to this theme. These findings indicate that some participants believe literary texts can help students develop their aesthetic appreciation and emotional awareness. In this context, P15 notes that students start to see language not just as a technical tool but also as an aesthetic structure through literary texts, stating: “I think they improved because they realized that language skills are not just about memorizing words, but that they can be expressed more beautifully through poetry.” However, it is important to highlight that this contribution was emphasized more by a smaller group of participants compared to other themes discussed in the study.

Data related to the theme of “Language Skills” are displayed in Table 7. The sub-theme “Contribution to Language Skills” within this theme captures participants’ statements on the role of literary texts in students’ language development. In terms of code frequency, this theme accounted for 8.10% of all codes and was mentioned 23 times. Seventeen teachers, representing 56.70% of participants, stated that literary texts helped students develop skills such as grammar, vocabulary, and communication. Regarding this, P25 said that literature contributes to both language development and students’ cognitive processes: “Literature helps our children think more deeply, and of course, literature is an abstract subject in a way. The effective use of language in literature is beneficial for both language and cognitive development.” However, only one teacher (3.30%) stated that literary texts did not contribute in this area. P23 expressed this view as follows: “It does not contribute much in terms of language skills because learning a language is unfortunately very difficult in our country, and this applies to both English and Arabic, but there is one thing: it broadens children’s horizons, so even if they cannot express themselves on a topic, even if they cannot form sentences in the language, even if they cannot construct sentences in Arabic very correctly, at least they have information about that person or event.” This situation shows that most teachers see literature as a valuable resource in language teaching.

Additionally, participants’ observations on how literary texts influence students’ interest and academic success are noteworthy. According to Table 8, the sub-theme “Student Interest and Participation” within the “Interest and Success” theme has 27 code occurrences (9.50%), with 14 participants (46.70%) sharing their views. This emphasizes the role of literary content in boosting motivation in the classroom. Participant P19 noted students’ strong interest in narrative genres by saying: “Students are particularly interested in stories, so we try to make sure that they are aware of them by mentioning them in class, thereby encouraging them to read.” Similarly, P17 discussed the personal connection students form with texts, explaining: “For example, when you say something about a biography author, they like it, they research it, they read the book, they learn something from it, and then they say to you, ‘Look, I researched what you said last time, and I found this book, and it says this and that. The sub-theme “Interest-Performance Relationship” also appears with 16 code instances (5.60%), and 11 participants (36.70%) believe that literature directly impacts students’ academic achievement. For example, P14 stated: “…his ability to express himself has changed, his self-confidence has increased, he gives clearer answers to my questions, he says ‘I can do it, I can say it,’ I think he has been influenced even just by reading poetry, and I think taking on a role, performing in a play, definitely influences him and impacts his academic performance.” It has been observed that literary content in classroom activities not only promotes cognitive development but also supports students’ emotional growth.

Figure 1 displays the distribution of themes based on participant views and code counts. The highest frequency is seen under the theme of “Use of Literary Texts” (T2) (*n* = 93). This shows that participants most often talked about using literary texts as teaching materials. Themes like “Cognitive Development” (T4), “Interest and Achievement” (T7), and “Literary Knowledge” (T3) were also frequently coded, indicating that teachers are becoming more aware of these topics. Conversely, the theme of “Literary Sensitivity” (T5) had the lowest frequency, suggesting that participants paid less attention to the emotional and aesthetic sides of literature. Differences in theme frequencies provide helpful insights into what participants associate with literature and literary practices; they demonstrate that teachers share their experiences and attitudes about the educational value of literature more clearly.

## 5 Discussion

The findings of this study show that teachers’ engagement with literature goes beyond language development, impacting their professional identity, pedagogical thinking, and interpretive skills. Teachers’ personal reading backgrounds and key literary experiences strongly shape their professional views and values in language education. The link between early literary experiences and professional growth suggests that teacher thinking and identity develop through continuous literary engagement, supporting Wenger’s (1999) view of identity as a dynamic, socially constructed concept. Emotional involvement in reading boosts teachers’ sense of professional belonging and reflective thinking. This aligns with research by Teng (2017) and Truong et al. (2025), who highlight the connection between emotions and self-confidence in professional development. As a result of this internalization, teachers’ perceptions of literature tend to be practical and goal-oriented.

Teachers’ perceptions of literature mainly focus on a practical approach: most participants highlighted its role in improving language skills, communication, and academic success. However, the data also show that engaging with literature helps develop critical skills -combining interpretive sensitivity, ethical reflection, and aesthetic appreciation. Participants who frequently used literary texts said they not only expanded students’ vocabulary but also enhanced their interpretive and cultural understanding. This dual focus supports Gao et al. (2024) model of teacher cognition, where cognitive and emotional factors work together to influence teaching decisions. It also emphasizes the CEFR’s focus on literature to build linguistic, cultural, and evaluative skills. Therefore, while curriculum constraints might cause teachers to emphasize measurable language outcomes, their continuous focus on empathy, imagination, and moral awareness reflects a broader view of language education that combines humanistic values with communication skills.

Text selection practices also reveal the link between professional competence and cultural literacy. Teachers’ choices reflected both pedagogical concerns, such as language level and lesson goals, and broader sociocultural orientations. Turkish language teachers tended to favor local or national canonical texts, while English language teachers emphasized Western authors. This pattern shows how literary prestige, educational traditions, and global standards influence reading habits and classroom discussions (Gao et al., 2024; Guerra and Wubbena, 2017). These differences suggest that teachers see literature not just as a linguistic tool but also as a symbol of cultural identity. The findings highlight the importance of teacher autonomy and cultural agency, revealing that literary preferences reflect not only instructional strategies but also identity formation. As Bardach and Klassen (2021) emphasize, autonomy and intrinsic motivation are key parts of professional dedication. In this study, teachers’ intrinsic motivation was clear in their meaningful use of literature as both a linguistic and ethical resource. Similarly, combining literary competence with pedagogical creativity helped develop innovative teaching methods.

At the core of these dynamics is teachers’ ability to connect their literary skills with pedagogical creativity. Participants described how reading literature enhanced their expressive clarity, interpretive flexibility, and classroom innovation. This connection is illustrated in the conceptual model developed in this study, which traces the progression from personal literary experience to professional skill. The model demonstrates how consistent reading habits foster reflection, empathy, and critical thinking, which are fundamental components of teacher cognition and professional identity (Wang and Yuan, 2025; Toköz, 2023; Sun, 2023). From this standpoint, and in line with the approach of Jiménez Pérez (2023), we distinguish critical thinking from critical competence; critical competence denotes a broader construct that additionally encompasses the capacity to translate such critical thinking into context-sensitive decisions, positions, and actions.

In this way, literature functions not only as a teaching resource but also as a reflective framework that encourages teachers’ growth as interpretive professionals, promoting dialogue and creative learning environments.

Teachers’ reflections highlight the broader educational benefit of engaging with literature: working with literature boosts students’ critical and creative thinking and enhances teachers’ reflective practices. Participants’ observations of improvements in analytical reasoning, imagination, and emotional expression demonstrate that literature promotes higher-level thinking in both students and teachers.

The study’s results align with earlier research highlighting the role of literature in promoting critical literacy and creativity through interpretive discussion (Sasson et al., 2018; Luukka, 2023). Overall, the findings depict literature as a multidimensional concept that combines cognition, emotion, and ethics, underlining its transformative role in language education and teacher development (Martel, 2015).

## 6 Conclusion

This study deepens understanding of how teachers’ literary experiences influence their professional identity, self-awareness, and instructional creativity. By integrating personal reading histories with teaching practices, it proposes a new framework connecting literary skills to teacher cognition and professional agency. The model shows how ongoing literary engagement enhances critical skills-such as interpreting, evaluating, and making language learning more human through empathy and creativity.

The study’s uniqueness lies in linking teachers’ experiential and cognitive aspects within a single conceptual model of literature-based professional development. This view enhances the discussion on teacher identity by showing that literary engagement acts as both a source of language development, ethical insight, and pedagogical innovation.

In practice, the findings highlight the importance of teacher education programs that include ongoing literary engagement, reflective reading, and text-based teaching in both pre-service and in-service training. Activities like collaborative reading circles and literary discussion workshops link personal growth with instructional practice, helping teachers become literary role models in the classroom.

Future research should examine this model across a wider variety of cultural and linguistic settings, employing longitudinal or mixed-method approaches to understand how teachers’ relationships with literature develop over time. Additionally, exploring how digital and generative AI tools affect reading habits and critical thinking could uncover new ways to improve literary skills in modern language education. By connecting personal literary experiences to pedagogical creativity, this study highlights literature’s continuing importance as a catalyst for reflective, ethical, and innovative teaching.

## 7 Limitations and recommendations

While the current study provides valuable insights, it also has some limitations. The sample was limited to Turkish and English language teachers, which limits how broadly the findings can be applied. Future research should expand to include teachers of different languages and educational systems. Additionally, relying on self-reported data introduces some subjectivity; to increase validity, interviews should be supplemented with classroom observations, and student perspectives should also be considered. Longitudinal studies could provide a deeper understanding of how teachers’ literary engagement and critical skills develop over time.

Pedagogically, teacher education curricula should emphasize critical reading, text selection, and discussion strategies that promote both linguistic and ethical awareness. In-service programs could incorporate reflective reading exercises and collaborative learning to improve teachers’ literary skills. Finally, future research should explore how AI-supported reading and writing environments affect teachers’ engagement with literature and their evolving teaching identities.

## Data Availability

The original contributions presented in the study are included in the article/supplementary material, further inquiries can be directed to the corresponding author.
